# Prevalence and potential determinants of chronic disease among elderly in India: Rural-urban perspectives

**DOI:** 10.1371/journal.pone.0264937

**Published:** 2022-03-11

**Authors:** Arup Jana, Aparajita Chattopadhyay

**Affiliations:** Department of Population & Development, International Institute for Population Sciences, Mumbai, Maharashtra, India; Population Council, INDIA

## Abstract

Chronic diseases are the leading causes of disability and premature death among the elderly population in India. The study, using data from the 75^th^ round of the NSSO survey (N = 44,631), examined the prevalence and determinants of chronic diseases among the population aged 60+ in India by applying bivariate and logistic regression analyses and used a non-linear decomposition technique to understand the urban-rural differences in the prevalence of chronic diseases. About 21% of the elderly in India reportedly have at least one chronic disease. Seventeen percent elderly in rural areas and 29% in urban areas suffer from a chronic disease. Hypertension and diabetes account for about 68% of all chronic diseases. The prevalence of chronic diseases is the highest in Kerala (54%), followed by Andhra Pradesh (43), West Bengal (36), and Goa (32). Those with higher levels of education, staying in urban areas, those who are economically dependent on others, staying alone or without spouse and children, and belonging to wealthy households have a higher likelihood of having a chronic disease. The probability of having a chronic disease is 1.15 times higher among urban residents as compared to their rural counterparts. Elderly rural women, compared to elderly rural men, and never-married, widowed, and divorced elderly urban women, compared to married elderly urban men, are significantly more likely to suffer from chronic ailments. Differences in education, wealth status, and caste are the three most significant contributors to the urban-rural gap in chronic diseases. The high risk of chronic diseases among certain subsets of the elderly population must be recognized as a key public health concern. The findings of our study will likely help promote healthy ageing in India.

## Introduction

The world’s population aged 60 and over is projected to reach around 2.1 billion by 2050 [1]. The rapid growth of the elderly population is adding to the burden of medical costs and compounding the shortage of service providers across the world [2]. Social change, unplanned urbanization, an unhealthy physical environment, and an unhealthy lifestyle are key drivers of chronic disease [3]. In low- and middle-income countries, chronic diseases account for 80% of all deaths [4]. The elderly population in India is expected to grow to 158.7 million by 2025, which would be 11.1% of the total population [5]. Over the past five decades, India has undergone a spectacular demographic transition, with the southern states becoming the biggest drivers of ageing. Many other states are experiencing the “elder boom,” mostly in rural areas [6]. According to the 2011 Census, more than 70% of India’s elderly population lives in rural settings [7]. Since chronic diseases are common in the elderly and affected by the surrounding environment and lifestyle [3], it is important to understand the prevalence and determinants of chronic diseases in urban and rural India.

India is currently experiencing a substantial burden of non-communicable diseases and lifestyle-related ailments [8, 9]. The rural elderly are mostly of lower socioeconomic status (SES) and highly dependent on others [9]. On the other hand, the urban elderly experience social exclusion, crime, and mental stress [10, 11]. While chronic diseases are dramatically rising across the country, there exists a significant socioeconomic and health inequality between urban and rural India, which is causing the urban-rural gap to widen [12]. People in urban areas have higher incomes and lead a more sedentary lifestyle than their rural counterparts [13, 14]. Besides, there are differences in types of jobs, education, wealth, social security, and health behavior, all of which are significant determinants of chronic diseases [15–18].

As a leading cause of premature death and disability at older ages, chronic diseases adversely affect a country’s Gross Domestic Product (GDP) [13, 19, 20]. Cardiovascular disease (CVD), diabetes, hypertension, cancer, and chronic respiratory diseases together formed around 60% of all the factors responsible for deaths in India in 2014 [21]. About 27% of Indian adults suffer from cardiovascular disease and 18% are diagnosed with diabetes, with the prevalence being much higher in urban areas as compared to rural areas [22]. Around 10% people living in rural areas have no access to essential medicines and only 19% have a health insurance [23].

In India, one in four people are likely to die of a chronic disease [24]. It is important, therefore, for adults and older persons to be aware of the dangers and adopt a healthy lifestyle for healthy ageing. The Indian public health system, which follows the framework of maternal and child health [25], is not fully prepared to cater to the health needs of the elderly population [26]. Since 1999, there have been various public policies and programs in place to improve elderly health, including the National Program for Health Care of the Elderly (NPHCE). And yet, nearly 50% of the elderly still need coverage [27]. Furthermore, India is going through a rural-urban convergence of non-communicable diseases [28]. Therefore, prioritizing health services in rural areas as much as in urban areas has become a concern for policymakers. Studies on chronic disease among the elderly are few in number and are mainly hospital-based, with a small sample size. Besides, not one study has examined this issue using the National Sample Survey (NSS) data of the Government of India. Our research is unique in its approach because it uses the NSS data to understand the prevalence and determinants of chronic disease among the Indian elderly and examines the contributors to the rural-urban differences. The findings of our study may help India achieve the SDGs aimed at promoting healthy living among the elderly.

## Data and methods

### Data

The study used unit-level data from the 75^th^ round of the National Sample Survey (NSS), which was carried out from July 2017 to June 2018. The National Sample Survey is responsible for conducting large-scale nation-wide household surveys on various socioeconomic subjects across India. For the 75^th^ round of the survey, a multistage stratified systematic sampling design was adopted. The first stage unit (FSU) was census village in the rural sector and Urban Frame Survey (UFS) block in the urban sector from which the households were selected. The survey was carried out in all the states and UTs of India, covering 1,131,823 households and 555,114 individuals with the respondents’ consent. A total of 8,077 villages and 6,181 urban blocks were selected randomly for the survey. Information on self-reported ailments (either diagnostic and / or symptomatic) was collected for a 15-day reference period and coded into 63 sections. A total of 237 was the missing sample in the study. A total of 93,925 cases of chronic diseases were reported in the study. Eight percent of all the survey participants, that is, 44,631 persons were aged 60 years and above, of whom 22,802 were men and 21,829 were women.

### Predictor variables

The associated factors of chronic disease were selected based on their inclusion in the published literature and the availability of the variables in the data set. Monthly per capita consumption expenditure (MPCE) quintile and economic dependency were two of the economic variables included in the study. The NSS had collected data on expenditures on food and non-food items. Data on food expenditure was collected based on a reference period of seven days, while that on non-food expenditure was collected based on reference periods of 30 days and 365 days. For our study, we standardized the food and non-food expenditures to a 30-day reference period [29]. Monthly per capita consumption expenditure was computed and used as the summary measure of consumption. It was then classified as Poor, Middle, and Rich. Age, sex, and marital status of the respondents were taken as demographic characteristics in the analysis. The socioeconomic variables incorporated in the analysis were place of residence, education, caste, religion, and living arrangement. The description of the selected variables can be seen in **Table 1**.

**Table 1:** Sample characteristics and classification of variables used in the analysis.

Variables	Response categories	Sample	Percent	Description of variables
Chronic disease	No	34,650	77.64	Having any chronic disease was recoded as Yes and No
	Yes	9,981	22.36	
Place of residence	Rural	24,436	54.75	Place of residence was used original
	Urban	20,195	45.25	
Sex	Men	22,802	51.09	Sex of the respondents was used original
	Women	21,829	48.91	
Age	60–64	22,603	50.64	Age of the respondents at the time of survey in completed years
	65–70	10,612	23.78	
	70+	11,416	25.58	
Marital status	Currently married	30,544	68.44	Marital status of the respondents was categorized as Currently married and Never/widowed/divorced (Never married, widowed/divorced)
	Never/widowed/divorced	14,087	31.56	
Education	Illiterate	21,292	47.71	Education was recoded as Illiterate (No literate), Primary or Below (literate without any or formal schooling, literate through TLC/ AEC or others and below primary; formal schooling), Middle/higher Schooling (primary, upper primary/middle, secondary, higher secondary) and Diploma/graduate & above (diploma /certificate course; graduation and above)
	Primary or Below	9,295	20.83	
	Middle/higher Schooling	10,100	22.63	
	Diploma/graduate & above	3,944	8.84	
Caste	Schedule Caste	3,954	8.86	Caste of mother was used as original
	Schedule Tribe	6,278	14.07	
	Other Backward Class	17,280	38.72	
	Others	17,119	38.36	
Religion	Hindu	10,232	22.93	Religion of mother was used as dummy variable in three groups from original variable. Others include (Sikh, Buddhist/Neo-Buddhist, Jain, Jewish, Parsi/Zoroastrian, No-religion, Donyi polo, Other).
	Muslim	17,280	38.72	
	Christian	17,119	38.36	
	Others			
Living arrangement	Only Spouse	4,866	10.9	Religion of mother was used as dummy variable in three groups; only spouse, With spouse and children (living with spouse and others or living with children and others) and Others (living alone or others or old age home or other relatives)
	With spouse and children	25,463	67.96	
	Others	14,302	21.14	
MPCE quintile	Poor	12,967	29.05	MPCE created as dummy variable in three groups from original variable. It is as Poorer + poorest = Poor, Richer + Richest = High
	Middle	14,215	31.85	
	Rich	17,449	39.1	
Economically dependent	Do not dependent	12,880	28.86	Economically dependent was used as original
	Partially	10,951	23.73	
	Fully	21,160	47.41	
	Total	44,631		
		71.32		

### Outcome variable

Elderly aged 60 years and above were included in the analysis. Self-reported morbidity status was used as the outcome variable. To determine the state of disease, respondents were asked to respond to the following question: "*What ailment have you suffered from during the last 15 days*?" We A total of 63 ailments were reported in the survey. We identified the chronic ailments considering the classification given by ICD-10 [30]. Chronic disease was taken as a dichotomous variable, where 1 signified having a chronic disease and 0 denoted not having a chronic disease. Since chronic diseases cannot be reported without a medical assessment, self-reported morbidity can be considered diagnosed morbidity in this regard.

## Methods

The prevalence of chronic diseases by the characteristics of the study population was described using weighted percentages to ensure the actual representation of the prevalence at national and domain levels. Sample weights were used to estimate the prevalence rates and generalize them for the whole country. Bivariate analysis was employed to estimate the prevalence rate of chronic diseases per hundred elderly persons. Binary logistic regression was carried out to examine the effect of socioeconomic variables on the prevalence of chronic diseases. The predictor variables were selected based on their inclusion in the previous studies and their availability in the dataset. A logistic regression model was used to assess the determinants of chronic diseases among the elderly population in India by rural and urban residence. The Blinder–Oaxaca decomposition was used to explain the contributors to the urban-rural gap in the prevalence of chronic diseases [31, 32]. The entire statistical analysis was carried out using STATA, whereas ArcGIS was used to create a map showing the prevalence of chronic diseases.

## Result

The sample distribution given in **Table 1** shows that out of 44,631 individuals, 51% were men and 49% were women. About 55% of the sampled population was from rural areas, and the remaining 45% from urban areas. Nearly 29% of the respondents were poor, while 39% belonged to rich households. As much as 74% of the elderly population was economically dependent on others, whereas 23% was partially dependent. Almost 68% of the Indian elderly were living with their spouses and children.

### Prevalence of chronic diseases

**Fig 1:**
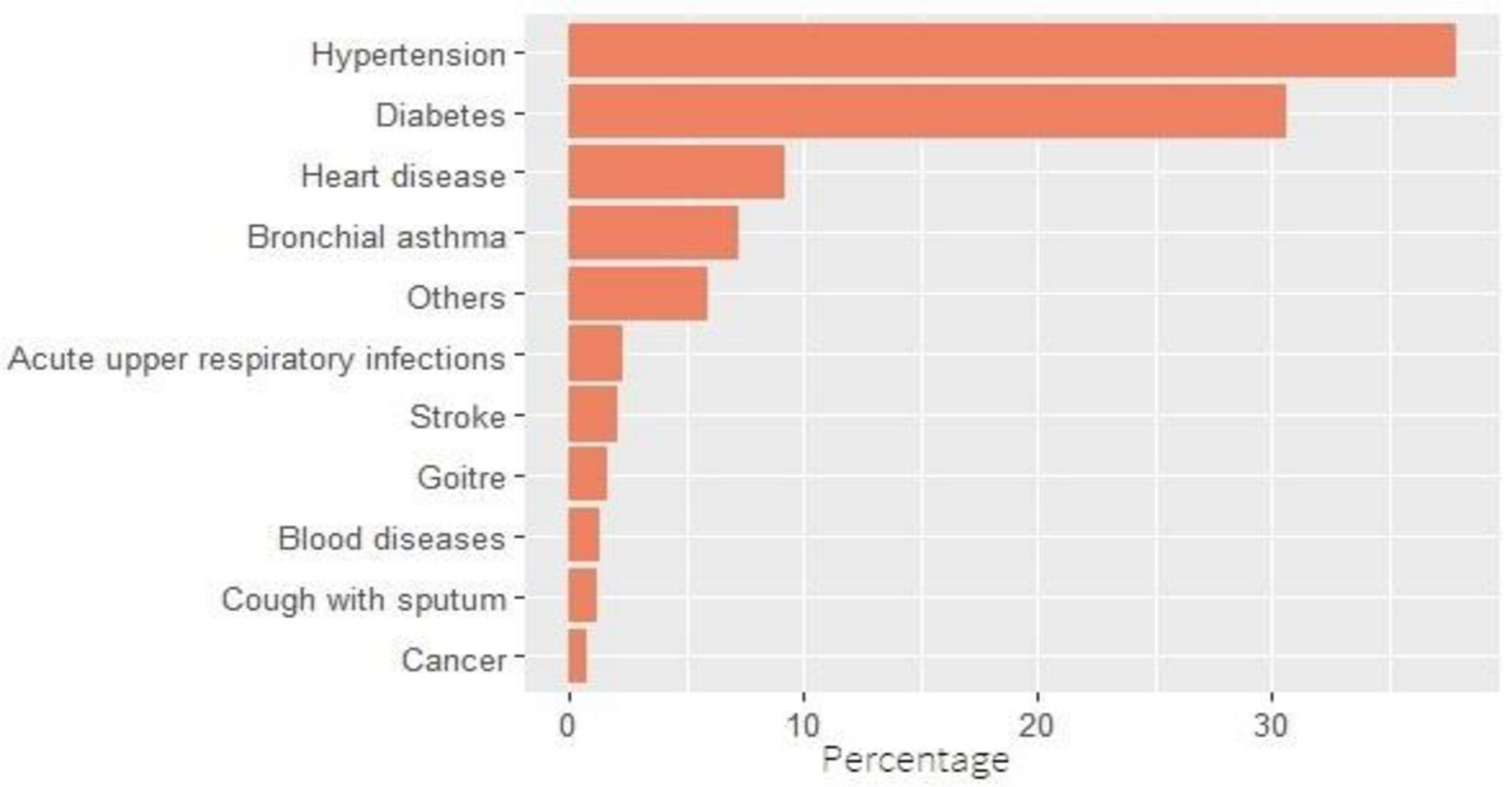
Percentage distribution of chronic diseases among ageing population, India, 2017–18 (based on 75th round of NSS).

**Fig 2:**
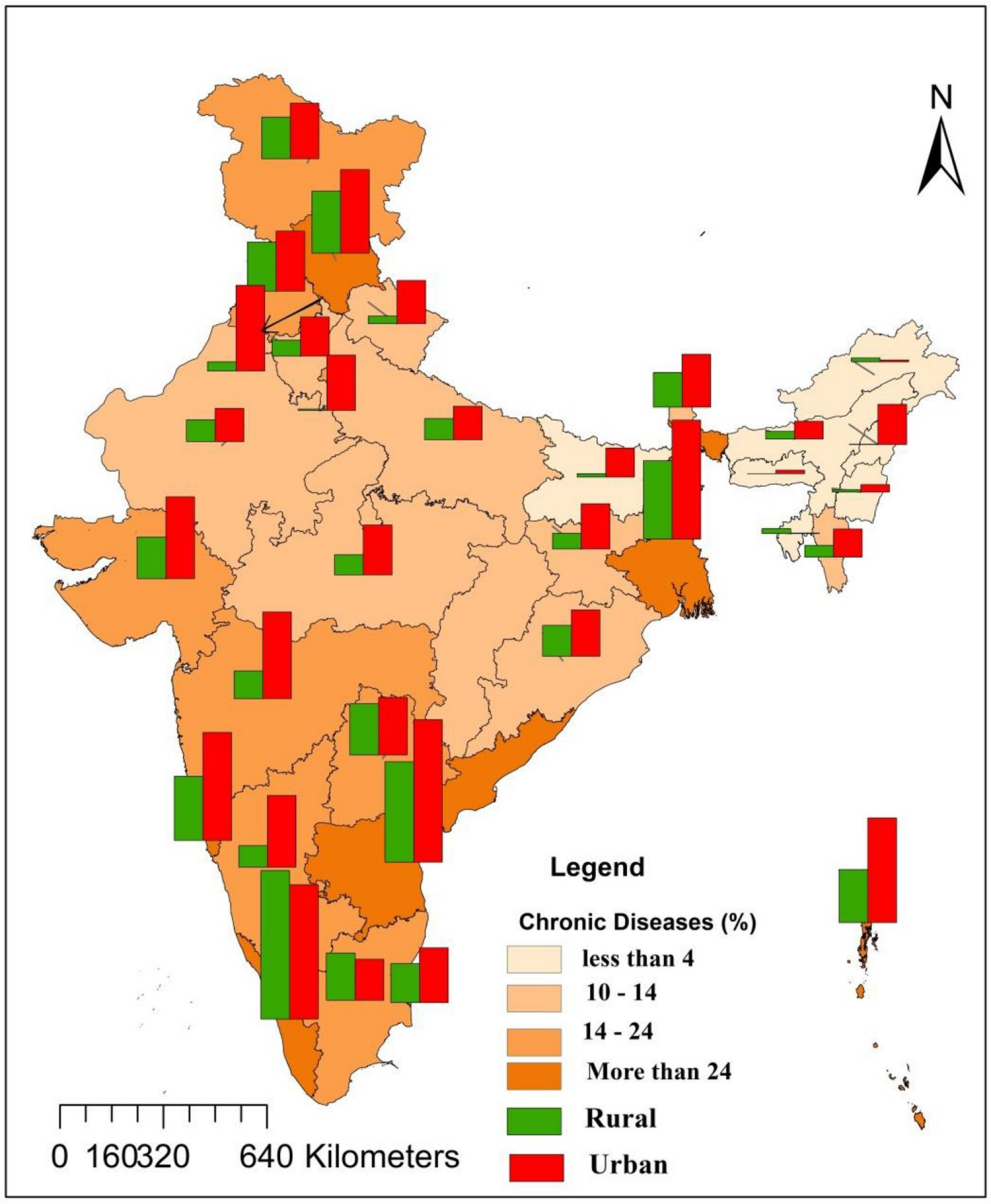
Prevalence map of chronic diseases among elderly population in India, 2017–18 (based on 75th round of NSS). Source: Prepared by authors.

**Fig 3:**
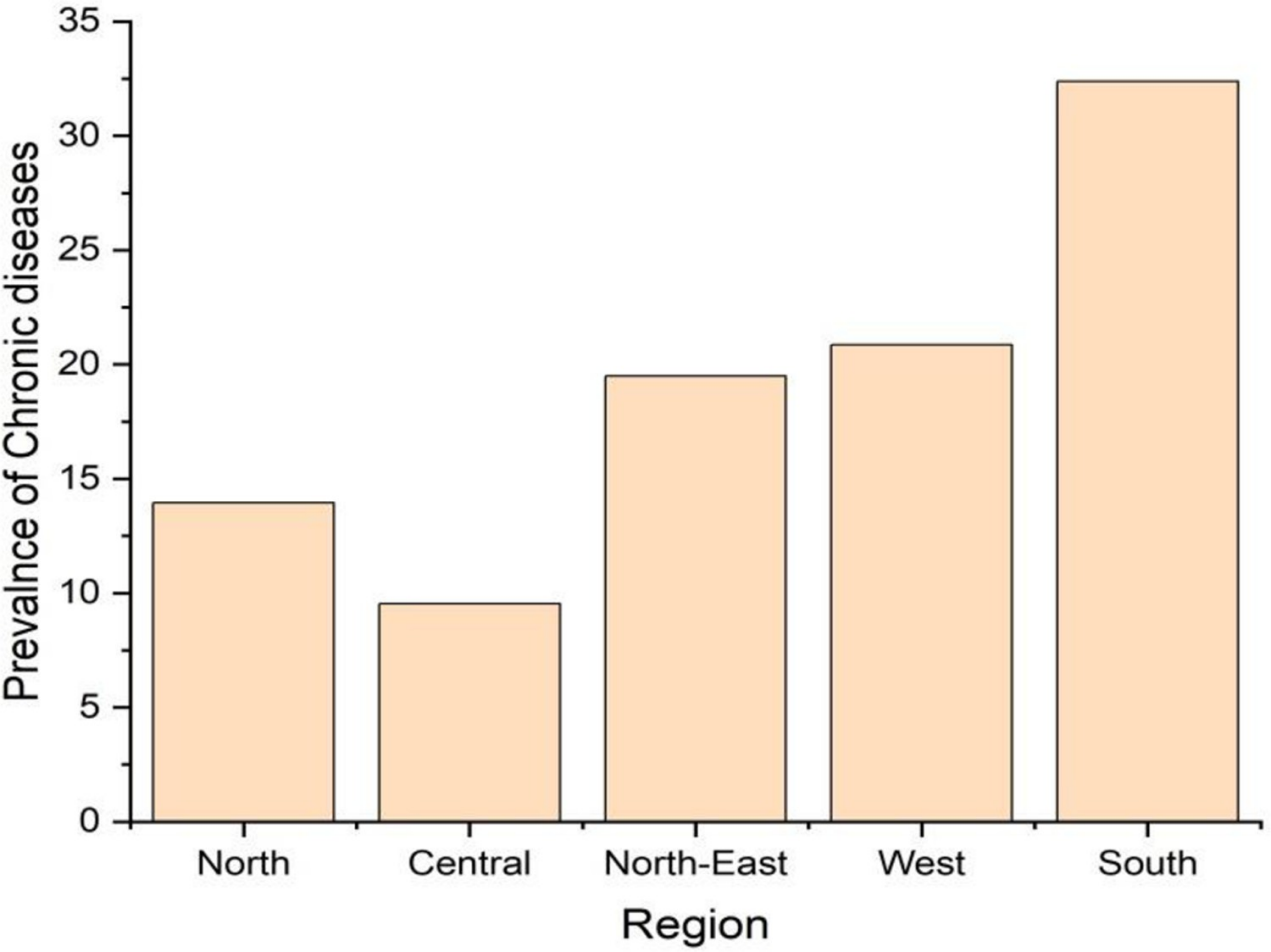
Region wise prevalence of chronic diseases among elderly in India, 2017–18 (based on 75^th^ round of NSS).

**Table 2** presents the prevalence of chronic diseases among aged people in India. The prevalence of self-reported chronic disease was 21 per 100 elderly Indian persons. Hypertension and diabetes were the most common diseases and accounted for 38% and 31% of all chronic diseases respectively (**Fig 1)**. Heart disease, acute upper respiratory tract disease, asthma, and stroke also had a considerable share. About 17% elderly were suffering from chronic diseases in rural areas as against 29% in urban areas. Except for Kerala and Tamil Nadu, in all the other states, the disease prevalence was higher in urban areas. A distinct rural-urban differential was observed in Maharashtra, Karnataka, Andhra Pradesh, and West Bengal. Among the states, the prevalence of chronic diseases was the highest in Kerala (54%), followed by Andhra Pradesh (43%), West Bengal (36%), and Goa (32%). Chronic diseases were less prevalent in the north-eastern states. Among the union territories, Lakshadweep (49%) had the highest prevalence, whereas Dadra and Nagar Haveli (1.4%) had the lowest prevalence. Regional differences in the prevalence were quite apparent (**Fig 2**). The highest prevalence of chronic diseases among the elderly was in the southern region (**Fig 3**) and the lowest in the central region.

**Table 2:** Prevalence of any chronic disease per 100 elderly population in India, 2017–18.

State	Rural	Urban	Total
Andaman & Nicobar Isl	20.39	40.23	28.43
Andhra Pradesh	38.65	54.78	43.08
Arunachal Pradesh	1.49	0.68	1.41
Assam	2.94	6.87	3.51
Bihar	1.31	11.2	2.74
Chandigarh	3.51	32.93	32.68
Chhattisgarh	8.40	9.28	8.59
Dadra & Nagar Haveli	0.00	13.19	1.48
Daman & Diu	7.09	-	6.02
Delhi	-	21.24	20.92
Goa	24.64	41.54	32.89
Gujarat	15.9	31.41	21.76
Haryana	6.14	15.02	9.77
Himachal Pradesh	23.82	32.13	24.51
Jammu & Kashmir	15.98	21.29	17.39
Jharkhand	6.03	17.37	8.87
Karnataka	8.20	27.47	15.87
Kerala	57.17	51.71	54.89
Lakshadweep	39.29	52.06	49.35
Madhya Pradesh	7.79	19.2	11.02
Maharashtra	10.6	33.25	20.39
Manipur	0.94	2.83	1.70
Meghalaya	0.00	1.32	0.38
Mizoram	4.52	10.8	7.60
Nagaland	0.07	15.42	3.39
Orissa	11.87	17.81	12.72
Pondicherry	14.98	21.07	18.52
Punjab	18.83	23.04	20.66
Rajasthan	8.36	12.72	9.36
Sikkim	13.32	20.3	13.58
Tamil Nadu	18.14	15.71	17.03
Telangana	19.74	22.12	20.74
Tripura	1.88	0.13	1.36
Uttar Pradesh	8.11	12.79	9.13
Uttarakhand	2.89	16.59	6.36
West Bengal	30.13	45.72	36.18
Total	16.66	28.72	20.68

Note:—small sample size

Source: Generated by authors based on NSS data

### Determinants of rural-urban gap in chronic diseases in India

The odds ratios (OR) obtained from the logistic regression analysis show the associated predictors of chronic disease among the elderly population in India (**Table 3**). The chances of having chronic diseases were influenced markedly by factors like sex, marital status, level of education, caste, religion, wealth, living arrangement, and economic dependency.

**Table 3:** Odds ratio and 95% Confidence Interval (CI) of any chronic disease associated with background characteristics by place of residence in India, 2017–18.

Determinants	Rural	Urban	Total
**Place of residence**			
Rural ®	-	-	1.00
Urban	-	-	1.15***(1.09, 1.21)
**Sex**			
Men ®	1.00	1.00	1.00
Women	1.07*(0.99, 1.16)	0.91**(0.84, 0.99)	0.98 (0.93, 1.04)
**Age**			
60–64 ®	1.00	1.00	1.00
65–70	1.17***(1.08, 1.27)	1.24***(1.14, 1.34)	1.20***(1.14, 1.28)
70+	1.56***(1.44, 1.70)	1.41***(1.31, 1.53)	1.48***(1.4, 1.57)
**Marital status**			
Currently married®	1.00	1.00	1.00
Never married/widowed/divorced	0.93 (0.79, 1.10)	1.22**(1.04, 1.43)	1.07 (0.96, 1.21)
**Education**			
Illiterate®	1.00	1.00	1.00
Primary or Below	1.92***(1.77, 2.09)	1.55***(1.41, 1.70)	1.77***(1.66, 1.88)
Middle/higher Schooling	2.07***(1.87, 2.28)	1.39***(1.27, 1.52)	1.68***(1.57, 1.79)
Diploma/graduate & above	2.2***(1.79, 2.71)	1.47***(1.31, 1.64)	1.72***(1.57, 1.89)
**Caste**			
Others®	1.00	1.00	1.00
Schedule Tribe	0.26***(0.23, 0.31)	0.48***(0.41, 0.57)	0.30***(0.27, 0.33)
Schedule Caste	0.53***(0.48, 0.59)	0.70***(0.63, 0.79)	0.55***(0.51, 0.59)
Other Backward Class	0.73***(0.68, 0.79)	0.79***(0.74, 0.85)	0.73***(0.69, 0.76)
**Religion**			
Hindu®	1.00	1.00	1.00
Muslim	1.75***(1.59, 1.94)	1.44***(1.31, 1.58)	1.6***(1.49, 1.71)
Christian	1.78***(1.57, 2.01)	1.31***(1.15, 1.49)	1.58***(1.45, 1.73)
Others	0.96 (0.81, 1.14)	0.82**(0.7, 0.97)	0.88**(0.78, 0.99)
**Living arrangement**			
With spouse and children®	1.00	1.00	1.00
Only spouse	2.15***(1.91, 2.41)	1.85***(1.66, 2.06)	1.97***(1.82, 2.13)
Others	1.39***(1.18, 1.64)	1.02 (0.87, 1.20)	1.18***(1.06, 1.33)
**Wealth index**			
Middle®	1.00	1.00	1.00
Poor	0.7***(0.64, 0.76)	0.83***(0.75, 0.93)	0.74***(0.69, 0.79)
Rich	1.19***(1.09, 1.30)	1.25***(1.16, 1.35)	1.21***(1.15, 1.28)
**Economically dependent**			
Fully®	1.00	1.00	1.00
Not dependent	0.74***(0.67, 0.81)	0.83***(0.76, 0.91)	0.79***(0.74, 0.85)
Partially	0.88***(0.81, 0.95)	0.86***(0.79, 0.94)	0.87***(0.82, 0.93)

Note: ® Reference category; Significant level

*** p < 0.001

** < 0.05

* p < 0.1

Source: Generated by authors based on NSS data

The study observed that urban residents had a higher probability of having chronic diseases (OR, 1.15; 95% CI, 1.09–1.21) as compared to rural residents. Interestingly, in urban areas, women were less likely to have chronic diseases (OR, 0.91; 95% CI, 0.84–0.99) as compared to men, while the scenario was just the opposite in rural areas, that is, men were less likely to have chronic diseases as compared to women (OR, 1.07; 95% CI, 0.99–1.16). The never married/widowed/divorced elderly had greater chances of having chronic diseases (OR, 1.22; 95% CI, 1.04–1.43) among urban residents. It is worth noting that the probability of having a chronic disease increased rapidly with age, with the odds among the 70+ elderly persons being 1.56; (95% CI, 1.44–1.70) and 1.41 (95% CI, 1.31–1.53) in rural and urban India, respectively as compared to those aged 60–64. The probability of having chronic diseases was much higher among the elderly with a comparatively higher educational attainment. For example, in rural areas, the odds of having a chronic disease among elderly persons having primary or below, middle or higher schooling, and diploma or graduate and above levels of education were 1.92; (95% CI, 1.77–2.09), 2.07 (95% CI, 1.87–2.28), and 2.20 (95% CI, 1.79–2.71) respectively. A similar trend was observed in urban areas. Muslim and Christian elderly, irrespective of their place of stay, were more likely to have chronic diseases as compared to their Hindu counterparts (OR, 1.78; 95% CI, 1.57–2.01). Compared to the “other” caste category, all social groups showed a lower probability of suffering from chronic illnesses. For example, in rural areas, the odds for Scheduled Caste (SC), Scheduled Tribe (ST), and Other Backward Class (OBC) elderly were 0.53 (95% CI, 0.48–0.59), 0.26 (95% CI, 0.23–0.31), and 0.73 (95% CI, 0.68–0.79) respectively. Similarly in urban India, the odds for these groups were (OR, 0.70; 95% CI, 0.63–0.73), (OR, 0.48; 95% CI, 0.41–0.57), and (OR, 0.79; 95% CI, 0.74–0.85) respectively. A significantly higher probability of having chronic diseases was observed among wealthy elderly (OR, 1.21; 95% CI, 1.15–1.28) against the middle class, while the odds were less among the poor (OR, 0.74; 95% CI, 0.69–0.79). Further, those who were economically independent (OR, 0.79; 95% CI, 0.74–0.85) were less likely to suffer from disease, when all other factors were controlled. In rural India, elderly persons living with others or alone (OR, 2.15; 95% CI, 1.91–2.41) and those living with only the spouse (OR, 1.39; 95% CI, 1.18–1.64) had higher chances of suffering from chronic diseases as compared to those who were living with their spouses and children. In this case, the odds were 1.85 (95% CI, 1.66–2.06) and 1.02 (95% CI, 0.87–1.20) respectively in urban areas.

After adjusting for socioeconomic and demographic variability, the disease-specific results (**Table 4**) obtained from the logistical regression show that older people in urban areas were more likely to have hypertension (OR, 1.31; 95% CI, 1.21–1.41), diabetes (OR, 1.64; 95% CI, 1.51–1.77), and respiratory infections (OR, 1.11; 95% CI, 0.96–1.31) than their counterparts in rural areas.

**Table 4:** Odds ratio and 95% Confidence Interval (CI) of top five chronic diseases associated with background characteristics in India, 2017–18.

**Determinants**	Hypertension	Diabetes	Heart Disease	Bronchial asthma	Upper respiratory infections
**Place of residence**					
Rural ®		1.00	1.00	1.00	1.00
Urban	1.31***(1.21, 1.41)	1.64**(1.51, 1.77)	1.02 (0.89, 1.16)	1.03*** (0.92, 1.11)	1.11 ***(0.96, 1.31)

Note: ® Reference category; Significant level

*** p < 0.001

** < 0.05

* p < 0.1; All socioeconomic and demographic variables have been adjusted for the model.

Source: Generated by authors based on NSS data

The Blinder–Oaxaca decomposition technique was employed to explain the gap in the prevalence between urban and rural areas. The method measures the proportion of discrepancy due to differences in the distribution of independent factors and also the part responsible for the differences in the influence of determinants between groups. The result of the decomposition analysis reaffirms that (**Table 5**) the prevalence of chronic diseases was lower among the elderly persons residing in rural areas. For instance, the probability of having chronic diseases was 0.18 among rural residents as compared to 0.27 among their urban counterparts. Education, caste, and wealth status were the most significant contributors to this gap. The highest contribution (43%) came from the educational attainment of the respondents, followed by their wealth status (32%), caste (23%), and age (4%).

**Table 5:** Contribution of factors in urban rural differentials in prevalence of chronic disease among elderly in India, 2017–18.

Contributing factors	Coef.	95% CI	Percentage Contribution
Mean prediction rural	0.1840 ***	(0.179,0.188)	
Mean prediction urban	0.2715 ***	(0.265,0.277)	
Difference (rural—urban)	-0.0875 ***	(-0.095, -0.079)	
Due to endowments	-0.04297 ***	(-0.049,0.037)	
Due to coefficients	-0.0153 ***	(-0.024,0.006)	
Due to interaction	-0.02926 ***	(-0.038,0.021)	
Total Explained	49.096%		
Sex	0.0000 ***	(-0.0001,0.0001)	-0.01
Age	-0.0017 ***	(-0.0024,0.001)	4.10
Marital status	0.0006 *	(-0.002,0.001)	-1.41
Education	-0.0185 ***	(-0.023, -0.014)	43.18
Caste	-0.0097 ***	(-0.001, -0.006)	22.65
Religion	-0.0000	(-0.0002, -0.000)	0.22
Living arrangement	-0.0006 **	(-0.001, -0.00)	1.45
Wealth	-0.0135 ***	(-0.187, 0.008)	31.57
Economic independency	0.0007 ***	(0.003, 0.001)	-1.77

Note: Significant level

*** p < 0.001

** < 0.05

* p < 0.1

Source: Generated by authors based on NSS data

## Discussions

The present study sought to assess the spatial variations in and the determinants of rural-urban differences in the prevalence of chronic diseases among the Indian elderly. Studies exploring national-level data on chronic diseases among elderly persons are minuscule in India. The study reveals that the overall prevalence of chronic diseases is about 21%, that is, 17% in rural areas and 29% in urban areas. Another study using data from the India Human Development Survey (IHDS) estimated a similar prevalence of chronic diseases among older Indian adults [23]. This reinforces that the estimates made in the present paper using NSS datasets are high quality. Interestingly, we could not find any other study that has used NSS data to estimate the prevalence of chronic diseases and its determinants in India. The recently published Longitudinal Ageing Study in India (LASI) also confirms that the Indian elderly are most vulnerable to chronic diseases such as hypertension, diabetes, and respiratory and heart disease [22]. Hence, we attempted to make use of this robust data set to understanding rural and urban prevalence and determinants of chronic disease.

The prevalence of chronic disease was observed to be the highest in Kerala, followed by Andhra Pradesh, West Bengal, Goa, and Uttarakhand. In urban areas, the prevalence was more in states like Maharashtra, Karnataka and Goa. These states have a higher consumption of tobacco and alcohol, a higher prevalence of obesity [33, 34], and a higher level of urbanization [35], all of which could be the possible reasons behind the higher prevalence of chronic diseases in these states [36].

Hypertension and diabetes account for about 70% of all the chronic diseases. As India is going through the third phase (Degenerative stress and man-made diseases) of epidemiological transition, chronic diseases such as diabetes and cardiovascular diseases are increasing rapidly with a rising proportion of the ageing population [37]. This, combined with the rising trend of obesity–especially in the southern region of India [38]–that triggers chronic diseases, is bothering the Indian policy makers [34].

Our study revealed that in urban areas, aged men had a higher probability of having chronic diseases in contrast to rural areas, where women showed higher odds of having chronic diseases. India accounts for 12% of the world’s smokers, of whom 50% are men [39]. Tobacco chewing and smoking [40], consumption of unhealthy food [41], and lack of physical activity [42] being more common among urban males are possibly why they have a higher risk of chronic diseases than urban females. On the other hand, in the countryside, elderly women are often subjected to neglect [43] and have more unmet health care needs [44, 45] than elderly men, leading to chronic diseases being more prevalent among them.

The study found that the likelihood of contracting chronic diseases increased with age, a result similar to that found in previous Indian studies [46]. The 70+ population in India has 40 to 60 percent higher likelihood of having a chronic disease as compared to the young old of 60–64 years. This underscores the urgent requirement of increasing the availability, accessibility, and affordability of health care services for India’s elderly population. Past studies have identified chronic cardiovascular and respiratory diseases as a leading cause of mortality in India [47]. As per the LASI report, about 37%, 11% and 10% elderly (aged 75 and above) reported suffering from cardiovascular diseases, diabetes. and lung disease respectively [22]. Recent study using LASI indicates that good health is the strongest predictor of work beyond age 60 and rural elderly’s economically gainful work heavily depends on health [48]. The Government of India launched the Ayushman Bharat program in 2017–18 to universalize health care facilities all over India [49]. Before that, the government had introduced the National Programme for Prevention and Control of Cancers, Diabetes, Cardiovascular Diseases and Stroke (NPCDCS) in 2010–11 to tackle Non-Communicable Diseases (NCDs) by installing NCD cells in all the districts of the country [50]. Another program, called the National Programme for the Healthcare of the Elderly (NPHCE), was launched to improve geriatric health care facilities. But the coverage of districts are less so far when the target was 325 districts [50]. Thus, universal coverage of the ongoing programs is urgently required, especially for the rural 70+ population, to contain the escalating burden of chronic diseases.

Having good family support can improve the mental and physical health of older adults [51]. The present study revealed that elderly persons living without family members had a lower chance of suffering from chronic diseases than those living with their families. Past evidence suggests that most elderly persons belonging to wealthy families and residing in urban areas live either alone or with only the spouse [22]. Living with family can be good for the older persons as the family can help them perform exercises, especially in rural areas where health facilities are poor. It has been observed in India that family members encourage the elderly people to engage in household chores, which helps them remain fit, a protective factor of chronic diseases [52]. Thus, family support and care play a major role in older people leading a healthy life in India. This observation is corroborated by another finding of this study that the never married/divorced/separated elderly persons are more prone to chronic diseases in urban areas. Previous studies have also found that living without the love and care of family and relatives is negatively associated with an older person’s health and quality of life [53–55]. Economically independent elderly is having lower likelihood of having chronic disease. This finding urges to create adequate job prospect to help elderly to get engaged in gainful work in context of poor social security.

Our study found a huge difference in the prevalence of chronic diseases by rural-urban residence. We also found that urban residence increases the risk of having three major chronic disease- i.e., hypertension, diabetes, and respiratory diseases. Higher education and more wealth are the most significant contributors to the urban-rural gap in chronic diseases among the elderly. Unhealthy food behavior, sedentary life, and a higher prevalence of obesity in urban areas may explain the gap [34, 35]. India has been experiencing a huge rural to urban migration for the last three decades due to better education and infrastructure and more and better employment and income opportunities in urban areas [55, 56]. However, urban areas are also characterized by sedentary lifestyle [34, 35], and unhealthy eating habits and diets [18, 19]. The Longitudinal Ageing Study in India report demonstrates that the urban elderly are physically more inactive than their rural counterparts [57–59] and experience more hypertension, diabetes, and obesity [19, 53, 60]. Another important driver of chronic diseases is air pollution, and urban residents are more exposed to pollutants than rural residents [61]. However, the possibility of worse healthcare facilities in rural areas leading to a lower likelihood of rural residents reporting chronic diseases cannot be discounted.

Caste too plays a vital role in explaining the higher prevalence of chronic diseases in urban areas. It is noteworthy that SC, ST and OBC respondents were found to be less likely to have chronic diseases as compared to their counterparts from the “other” caste category. Previous studies argued that higher caste people (non-SC/ST/OBC) have a higher income and standard of living and more access to sedentary hobbies (watching television, playing video games) and make greater use of vehicles for transportation [62, 63]. The diet pattern of SCs or STs is less risky for chronic disease [64] and they are more agrarian that demands more physical work. Past studies have also observed that most higher caste workers engage in “white collar” work as most of them live in urban areas [62, 63]. Underdiagnosis or underreporting of morbidity could be another reason behind the lower prevalence of chronic diseases among the SC and ST population.

The present study has some strengths as well as some limitations. The study estimated the prevalence of chronic diseases and their determinants from the urban-rural perspective. Very limited research has been done on this topic at the national level [23]. Our study fills this void by estimating the prevalence and spatial distribution of chronic diseases among elderly persons in the country. At the same time, we acknowledge the following limitations of the study. First, due to the unavailability of data, we were unable to investigate the role of risk factors such as physical inactivity, poor diet, and tobacco and alcohol consumption in explaining disease prevalence. Second, due to the cross-sectional nature of the data, it was not possible to establish a causal relationship.

## Conclusions

There is a dearth of studies on chronic diseases among the Indian elderly at the national level. This study explored that one in five elderly are suffering from chronic diseases in India. The prevalence of chronic diseases is much higher in urban than rural India. Higher levels of education, greater wealth, and dominance of ‘other’ castes in urban areas are the major contributors to the urban-rural gap in the prevalence of chronic diseases. Elderly men in urban areas and female in rural areas suffer more from diseases. Further research is needed to explore the gender differentials we found in this study. Keeping elderly economically active and encouraging them to have an active life style are perhaps a solution at this moment for India to delay the onset and tackle chronic disease. In addition, the present research would help policymakers to strengthen better health care services to the elderly population at state and national level for to achieving SDG targets. The research underscores the importance of strengthening eco-urban planning and developing better health programs, such as creating green spaces, making people aware of active life and providing economically meaningful service on an urgent basis to tackle chronic diseases. In India, family–especially spouse and children–are an excellent source of support for the elderly in terms of their health. Thus, developing community-based support and service provisions, in light of the dwindling family support in urban India, may be a critical measure to address chronic diseases. The WHO recently introduced the "Age-Friendly Primary Health Care Centres Toolkit" [64] to improve care quality. Hopefully, India with series of programmatic initiatives would reap the benefit of aging gracefully.
